# Hypothesis That Urethral Bulb (Corpus Spongiosum) Plays an Active Role in Male Urinary Continence

**DOI:** 10.1155/2016/6054730

**Published:** 2016-01-31

**Authors:** Peter Rehder, Nina M. Staudacher, Joerg Schachtner, Maria E. Berger, Florian Schillfahrt, Verena Hauser, Raphael Mueller, Viktor Skradski, Wolfgang Horninger, Bernhard Glodny

**Affiliations:** Department of Urology, Division of Reconstructive Urology, Medical University Innsbruck, 35 Anich Street, 6020 Innsbruck, Austria

## Abstract

The proximal urethral bulb in men is enlarged, surrounds the bulbous urethra, and extends dorsally towards the perineum. During intercourse engorgement takes place due to increased blood flow through the corpus spongiosum. Antegrade ejaculation is facilitated by contraction of the bulbospongiosus muscles during climax. Micturition during sexual stimulation is functionally inhibited. Supporting the bulb may indirectly facilitate continence in a certain subset of patients with postprostatectomy incontinence. During physical activity with increased abdominal pressure, reflex contraction of the pelvic floor muscles as well as the bulbospongiosus muscles occurs to support sphincter function and limit urinary incontinence. Operations to the prostate may weaken urinary sphincter function. It is hypothesized that the distal urinary sphincter may be supported indirectly by placing a hammock underneath the urethral bulb. During moments of physical stress the “cushion” of blood within the supported corpus spongiosum helps to increase the zone of coaptation within the sphincteric (membranous) urethra. This may lead to urinary continence in patients treated by a transobturator repositioning sling in patients with postprostatectomy incontinence. This paper describes the possible role of the urethral bulb in male urinary continence, including its function after retroluminal sling placement (AdVance, AdVance XP® Male Sling System, Minnetonka, USA).

## 1. Introduction

Postprostatectomy urinary incontinence still remains a significant problem in 2015. Of patients suffering from postprostatectomy urinary incontinence (PPI) roughly two-thirds have mild to moderate and one-third have severe urinary incontinence [1]. Worldwide about 200 million people suffer from urinary incontinence of all causes [2]. The incidence of urinary incontinence after prostate surgery (transurethral resection, simple open prostatectomy, and radical prostatectomy) varies between 1 and 5% [3]. Surgery to the prostate may lead to incontinence rates of up to 77% depending on definition and time after surgery [4]. Only a small proportion of patients with PPI are being treated with surgery. No randomized controlled studies exist comparing various operative techniques to treat male urinary incontinence. Recently a review paper has been published comparing different devices including the artificial urinary sphincter (AUS) AMS 800, Argus system (Promedon SA; Cordoba, Argentina), suburethral I-STOP TOMS (CL Medical), AdVance® Male Sling System (American Medical Systems, Minnesota, MN, USA), two-balloon ProACT*™* (Medtronic, USA), ATOMS® device (AMI, Vienna, Austria), ZSI 375 artificial urinary sphincter (Zephyr Surgical Implants, Geneva, Switzerland), novel remotely controlled, artificial urinary sphincter, Virtue quadratic sling (Coloplast, Humlebaek, Denmark), and the periurethral constrictor (Silimed) [5]. It was argued that all newer devices should be compared to the reported gold standard: the AUS AMS 800. This argument is based on the principle that the treatment of PPI functions on grounds of compression to the urethra and thus obstruction within the urethral lumen. The “functional” transobturator retroluminal repositioning sling supports sphincter function and* does not compress to obstruct* the urethral lumen. It functions as a dynamic hammock during moments of increased abdominal pressure [6–15]. The dilemma is that the mechanism of action of the “functional” sling is different to purely compressive slings or devices. A direct comparison is thus not possible, because compression versus support is like apples versus pears.

This paper tries to explain the role of the urethral bulb in urinary sphincter function, especially when being supported from below by a sling that indents the corpus spongiosum only. We believe that the role of a well vascularised urethral bulb is more important to urinary continence than thus far realised.

## 2. Materials and Methods

A short excursion is undertaken to describe the functional anatomy relevant to the urethral bulb, postprostatectomy incontinence (PPI), and diagnosis and workup of PPI and male urethral sling surgery. The current understanding of male urethral sphincter remains an area of constant debate. A PubMed and Google Scholar search was done to include some relevant publications in the discussion. A short description is included to demonstrate the surgery involved when placing a transobturator retroluminal repositioning sling. Images using magnetic resonance and perineal ultrasound illustrate final sling position and intact urethral bulb blood supply.

## 3. Results

### 3.1. Internal Urinary Sphincter (Lissosphincter, Smooth Muscle Fibres)

The so-called internal sphincter is composed of smooth muscle fibres and elastic tissues independent of the external sphincter, detrusor, and trigonum vesicae muscles. During prostate surgery this structure is irreparably damaged. Unmyelinated nerve fibres penetrate the smooth muscle layer at the level of the bladder neck and of the proximal part of the male urethra at 5 and 7 o'clock.

### 3.2. External Urinary Sphincter (Rhabdosphincter, Striated Slow-Twitch Muscle Fibres)

The so-called rhabdosphincter surrounds the sphincteric urethra in a horseshoe fashion. It is most pronounced anteriorly and anterolaterally, inserts on the distal ventral prostate capsule, and forms a dorsal midline raphe that fuses cranially with the posterior prostatic fascia/seminal vesicle fascia and caudally with the central perineal tendon. The orientation of the muscle fibres seems unclear as it depends on the three-dimensional orientation of the histological slices. The fibres are predominantly of the slow-twitch type which supports the notion of passive function. It is innervated by the inferior hypogastric plexus, which contains both sympathetic and parasympathetic fibres. These fibres run partly with the neurovascular bundle (NVB). The myelinated nerve fibres enter the striated sphincter on either side posterolaterally at 3 o'clock and 9 o'clock [17, 18]. The rhabdosphincter may be shortened lengthwise and be structurally damaged during radical prostatectomy.

### 3.3. Levator Ani and Puboperinealis Muscles (Sphincter Support, Striated Fast-Twitch Muscle Fibres)

The levator ani contains several parts and it is the innermost muscle of the pelvic floor. The bilateral puboperinealis muscles originate from the pubis and run laterally to the prostate and the sphincteric urethra to meet dorsally at the perineal body. Contraction of these mainly fast-twitch muscle fibres leads to angulation of the urethra with the resultant stop to urine flow. Damage to these structures during prostate apical dissection and also urethrovesical anastomosis may contribute to postsurgical urinary incontinence [17].

### 3.4. Supporting Structures of the Male Urethra

The pubovesical and the puboprostatic ligaments (=pubourethral ligaments) together with the tendinous arch of the pelvic fascia form the anterior urethral attachments to stabilize it behind the pubis. Denonvillier's fascia, the rectourethralis muscle, the perineal body, and the levator ani complex combine to form the posterior support to the sphincteric urethra. The double-layered Denonvillier's fascia covers the posterior surface of the prostate and seminal vesicles, thus separating it from the rectum. The function and existence of the rectourethralis muscle are unclear. The perineal body, also termed as central perineal tendon, borders ventrally to the bulb of penis and dorsally to the anal canal. Cranially it reaches the external urethral sphincter. Caudally and laterally the perineal body fuses with the tissues of the perineum and with the puborectalis muscle [18].

### 3.5. Human Visible Data Set

Brooks et al. produced an excellent paper demonstrating the male pelvic floor in relation to the urethral sphincter [18]. In summary, the levator ani muscles are vertically oriented. The urogenital hiatus reaches from the pubis to the perineal body. This means that the puboperinealis muscles and fascia play an important role during an increase of abdominal pressure to maintain continence. Rupture of these elements during prostate surgery may lead to the loss of dorsal support to the sphincteric urethra. Furthermore the anterior aspect of the rhabdosphincter reaches to* below* the level of the levator ani. The trigone seems continuous with the anterior fibromuscular stroma of the prostate. The thickest portion of the lissosphincter is within the prostate and tapers distally. Cranially from the apex of the prostate the striated urethral sphincter runs to the perineal membrane caudally. The anterior rhabdosphincter is nearly twice as thick as the posterior aspect, and the anterior length is also considerably longer than the posterior aspect. To preserve as much anterior rhabdosphincter as possible is important during radical prostatectomy.

**Figure 1:**
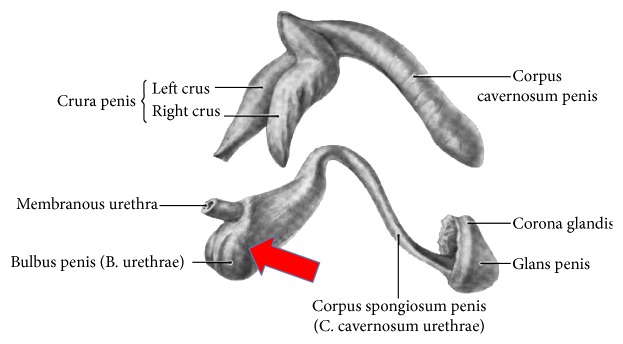
Anatomy corpora cavernosa and spongiosum: note the rather large urethral bulb. Red arrow shows direction and position of transobturator retroluminal repositioning sling (AdVance Male Sling System, American Medical Systems (Minnetonka, USA)). Adapted from Public Domain: Wikipedia, 15.4.2015 at 12h00, available from http://en.wikipedia.org/wiki/Corpus_spongiosum#/media/File:Grant_1962_198.png.

**Figure 2:**
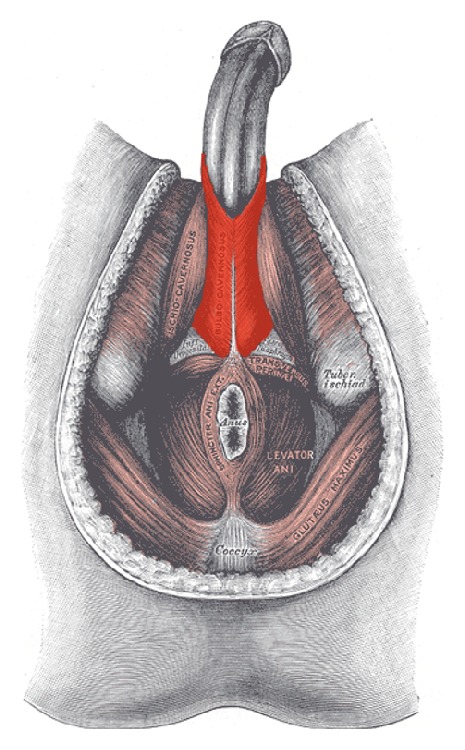
Bulbospongiosus muscle (red). Adapted from Public Domain: Wikipedia, 15.4.2015 at 12h00, available from https://en.wikipedia.org/wiki/Bulbospongiosus_muscle#/media/File:Bulbospongiosus-Male.png.

Figure 1 demonstrates the greater volume of spongeous tissue in the proximal corpus spongiosum, being surrounded by the bulbospongiosus muscle (Figure 2). Contraction of the bulbospongiosus muscle will increase the pressure within the corpus spongiosum which in turn will transfer the pressure wave onto the urethral wall. We believe that this mechanism is an important adjunct to the sphincter mechanism, especially when the proximal sphincter mechanism is compromised like after prostatic surgery.

### 3.6. Postprostatectomy Urinary Incontinence in Men

Major causes of stress incontinence (SUI) in men are radical prostatectomy (RP) and transurethral resection of the prostate (TURP). It may lead to intraoperative damage of the nerves, blood supply, and/or the urethral sphincter including its supporting structures. According to studies the incidence of SUI in men at 1 year after RP varies between 5% and 65% [4]. Postprostatectomy urinary incontinence may be brought about by changes to the urethra and sphincter as a direct result of the surgery. These changes were traditionally referred to as intrinsic sphincter deficiency. The effect of urethral closure, that is, continence due to sphincter function, is the combination of intact urothelium, nonscarred urethral wall, functioning smooth and striated muscle components of the sphincter, and the correct position of the sphincteric urethra with regard to the pelvic floor [15]. SUI in females is different as degrees of pelvic floor prolapse play a role. Typically the urethra is not directly damaged by birth or surgery. In recent years it turned out that the functional urethral length seems to be important for urinary continence. It should be more than 28 mm to maintain continence, ideally also after radical prostatectomy [19].

### 3.7. Diagnosis of Postprostatectomy Incontinence

The diagnosis of postprostatectomy incontinence (PPI) includes a history, physical examination, urinalysis, micturition diary, and questionnaires: International Consultation on Incontinence Questionnaire-Short Form (ICIQ-SF). The degree of severity of SUI is graded by the number of pads used per day and by a standardized pad test (1 or 24 hours). It is divided in mild (1 or 2 pads per day), moderate (3 or 4 pads per day), and severe SUI (more than 5 pads per day). Subsequently a urine flow measurement with postvoid residual, endoscopy, and video urodynamics complete the diagnostic workup.

**Figure 3:**
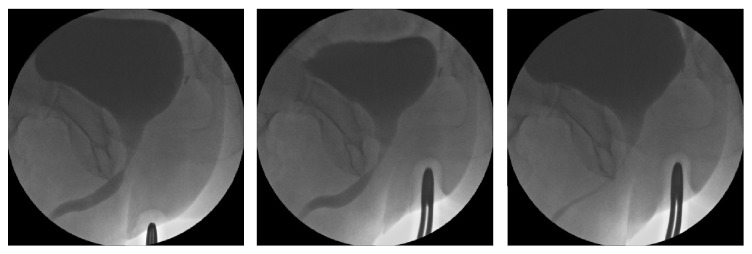
Midperineal elevation demonstrating prompt narrowing of the lumen within the sphincteric urethra. This test shows healthy sphincter reactivity. This test does not give an indication of length of sphincteric urethral coaptation. Images ^©^Peter Rehder.

For a functional sling implantation one of the most important examinations is a test utilizing midperineal support during dynamic urethroscopy and micturating cystourethrography (MCUG) to evaluate residual postsurgical urinary sphincter function. The patient is placed in the lithotomy position and the surgeon uses a cystoscope to look down the urethra. The midperineal area is digitally elevated by the examiner in a direction parallel to the membranous urethra to check for circumferential closure of the sphincter that is distinctly different from simple urethral closure by compression (Figure 3). An estimation of the length of functional closure maintaining passive elevation is done [11, 20]. The authors believe that better outcomes after sling insertion have been found in patients with a zone of coaptation/functional membranous urethral occlusion >1–1,5 cm [21]. This finding still has to be confirmed by prospective multicentre studies.

### 3.8. Operative Technique of AdVance/AdVance XP Sling Implantation (Figure 9)

The patient is put in the lithotomy position. The legs are bent 90 degrees to the horizontal and the knee width should be just wider than shoulder width. The legs are rotated slightly inward to relax the adductor muscles to facilitate easier trocar passage. For identifying the urethra during the implantation of the sling a 14 French (F) Foley catheter is placed. A midline perineal incision approximately 5 cm in length is made and the dissection carried though the subcutaneous tissue using electrocautery down to the bulbospongiosus (BS) muscle. The BS muscle is opened in the midline to expose the corpus spongiosum (CS), which is mobilised distally, laterally, and inferiorly up to the central tendon [22]. For adequate exposure of this region stay sutures or the Lonestar™ Retractor is used. The insertion location for the helical needle trocars is 1-2 cm below the implantation of the adductor longus tendon in the groin fold laterally to the ischiopubic ramus. The AdVance helical needle is held at a 45° angle to the midline incision and flushed onto the buttock [15].

**Figure 4:**
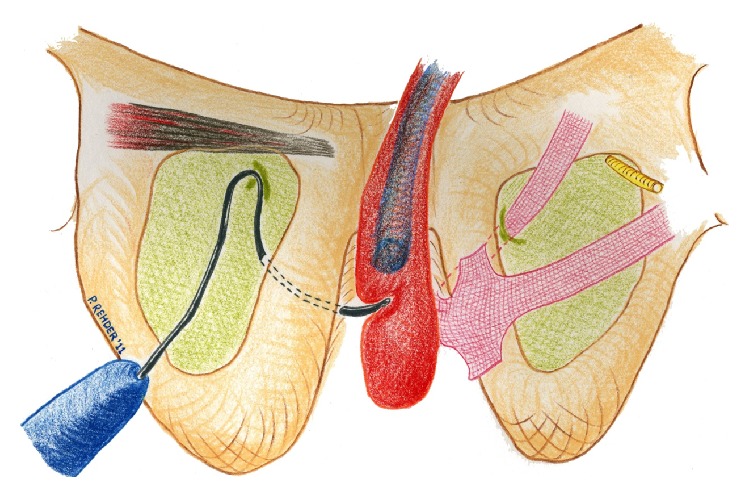
Correct placement of helical needle trocar from “outside-in.” The tip of the helical needle trocar enters the perineal wound underneath the lumen of the urethra in a distal position and underneath the membranous (sphincteric) urethra. The entrance of the introducer needle tip into the perineal wound should be in the uppermost corner between inferior pubic ramus and urethral bulb. The corpora cavernosa (not shown) lie “on top” of the inferior pubic rami and are not in the line of the needle trocar passage-measured safety margin of sling to dorsal penile nerve 5 mm. Note that the level of the tip of the trocar is and should be below the lumen of the membranous urethra. When the sling is tensioned, it is pulled into a straight line well underneath the level of the caudal membranous urethral wall. Only the distalmost portion of the membranous urethra is thus supported from the dorsal side. Correct sling placement should therefore have a very low risk of urethral erosion. The main risk for urethral damage is intraoperative perforation of the urethra during trocar passage. It is imperative to protect the urethra, noticed by a transurethral catheter in situ, with the surgeons' index finger, during needle trocar passage.

**Figure 5:**
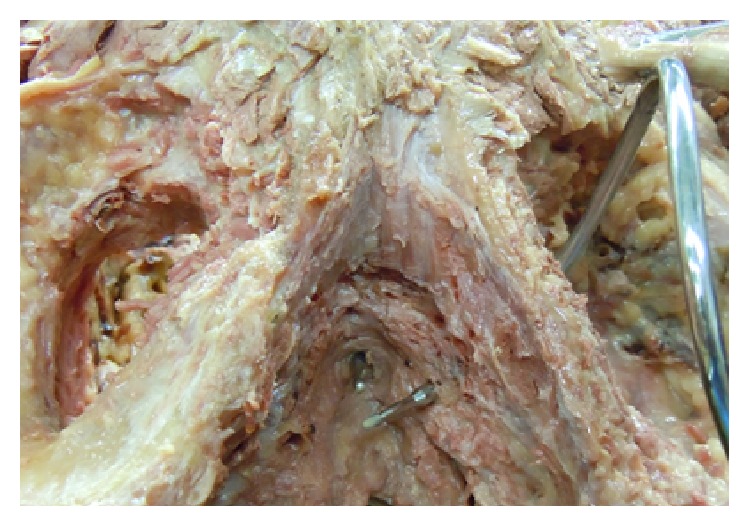
Cadaver dissection demonstrating helical trocar for transobturator route in relation to the sphincteric urethral lumen with bulb-headed probe in situ. Image, ^©^Peter Rehder [15].

**Figure 6:**
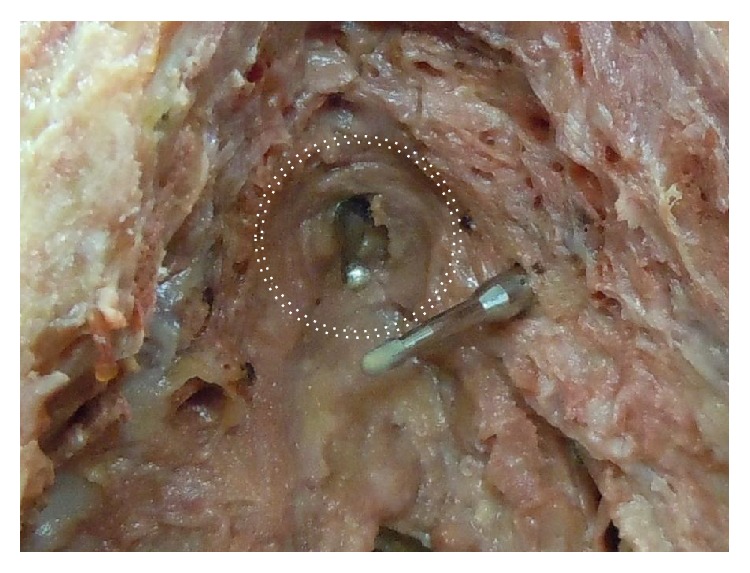
The dotted double circle is showing the extent of the urethral sphincter outer circumference. Note that the tip of the helical trocar is well below the sphincteric urethral lumen. Image, ^©^Peter Rehder [15].

**Figure 7:**
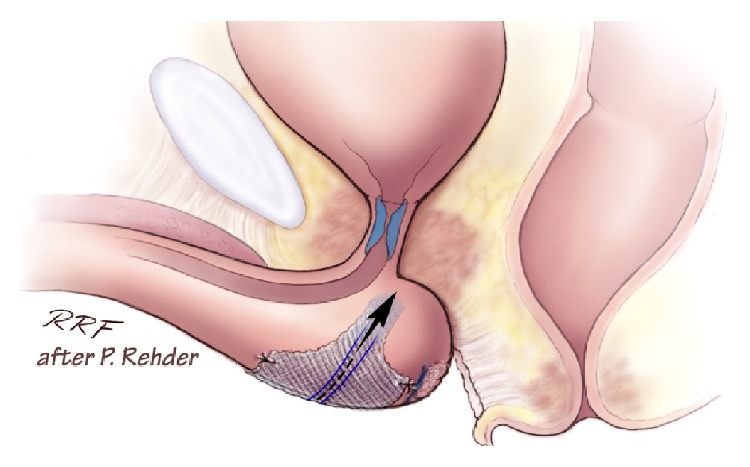
Positioning of sling at proximal urethral bulb before tensioning. Note relative short zone of coaptation, blue area within sphincteric urethra. Image, ^©^Peter Rehder [15, 16].

**Figure 8:**
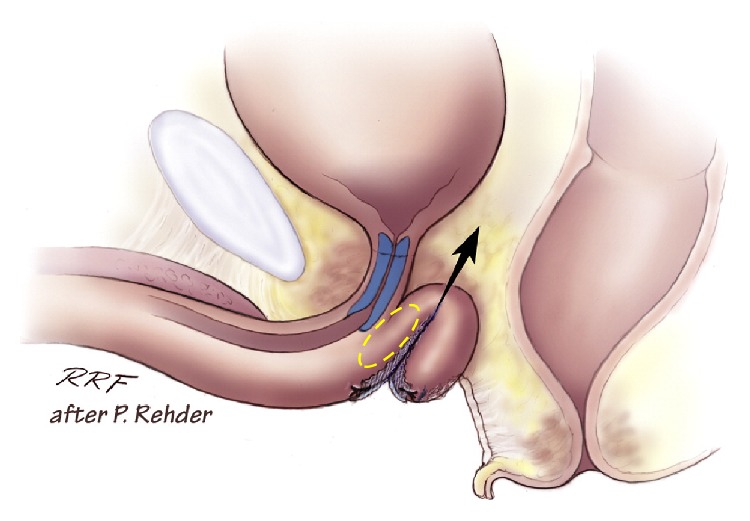
Indentation of the proximal corpus spongiosum by sling, distal and dorsal to the sphincteric urethra. Note longer zone of urethral coaptation marked in blue. Note the “cushion” of healthy spongeous tissue between the sling and the distal sphincteric urethra (yellow ellipse). Image, ^©^Peter Rehder [15, 16].

The index finger is placed in the incision below the ischiopubic ramus to protect the urethra and to guide needle placement. The AdVance needle is then driven straight in through the puncture site. Two or three pops are felt, and after the second or third pop the needle is rotated [15, 22]. When the incision point is properly selected, the needle goes through the obturator foramen without damage to other structures. Figures 4, 5, and 6 demonstrate the tip of the introducer trocar* below* the level of the distal membranous urethral lumen, including the corresponding sphincter fibres. One end of the sling is connected with the end of one trocar. Now the trocar is rotated backward to position the sling through the obturator fossa inside-out. On the contralateral side the same steps are repeated. With the helical trocar having being passed correctly outside-in both ends are pulled lightly to have the midportion touch the bulb slightly, without indenting the bulb. This is the correct position of the broader midportion. Only the “distal” edge needs to be fixed onto the bulb by 2 or 3 resorbable sutures. After that, the surgeon pulls on both arms of the sling at the same time to bring the sling in the right position. This results in a double fold of the mesh and an indentation of the bulb (Figures 7 and 8). Finally the surgeon completes it with subcutaneous tunneling of the sling arms to reduce the risk of sling slippage and thus loosening. After sling implantation a urethroscopy check is recommended. During the first twelve weeks after surgery the patient should refrain from heavy exercise or squatting. Follow-up is done according to local protocol. The procedure is rather easy to learn with no relevant differences in outcome according to learning curve [23]. Meticulous attention to detail remains important though.

### 3.9. Postoperative MRI Images

**Figure 10:**
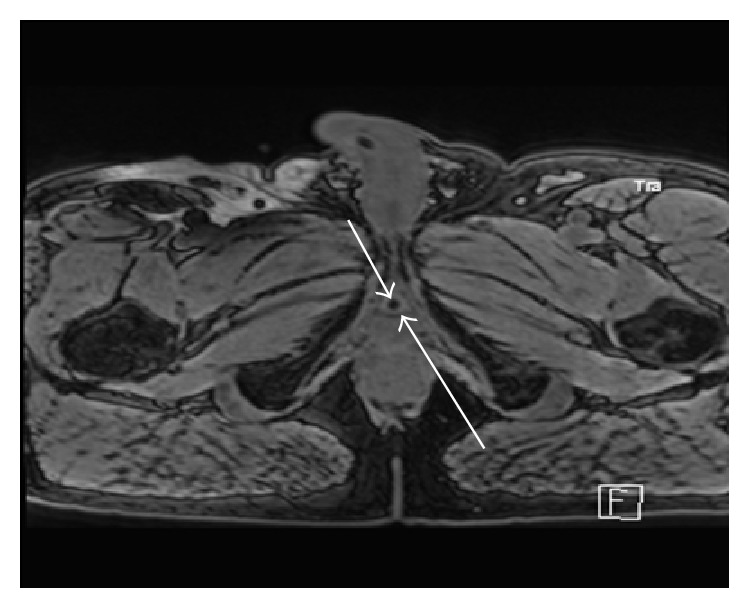
Retroluminal position of transobturator sling (long arrow) dorsal to the urethral lumen (short arrow).

**Figure 11 fig11:**
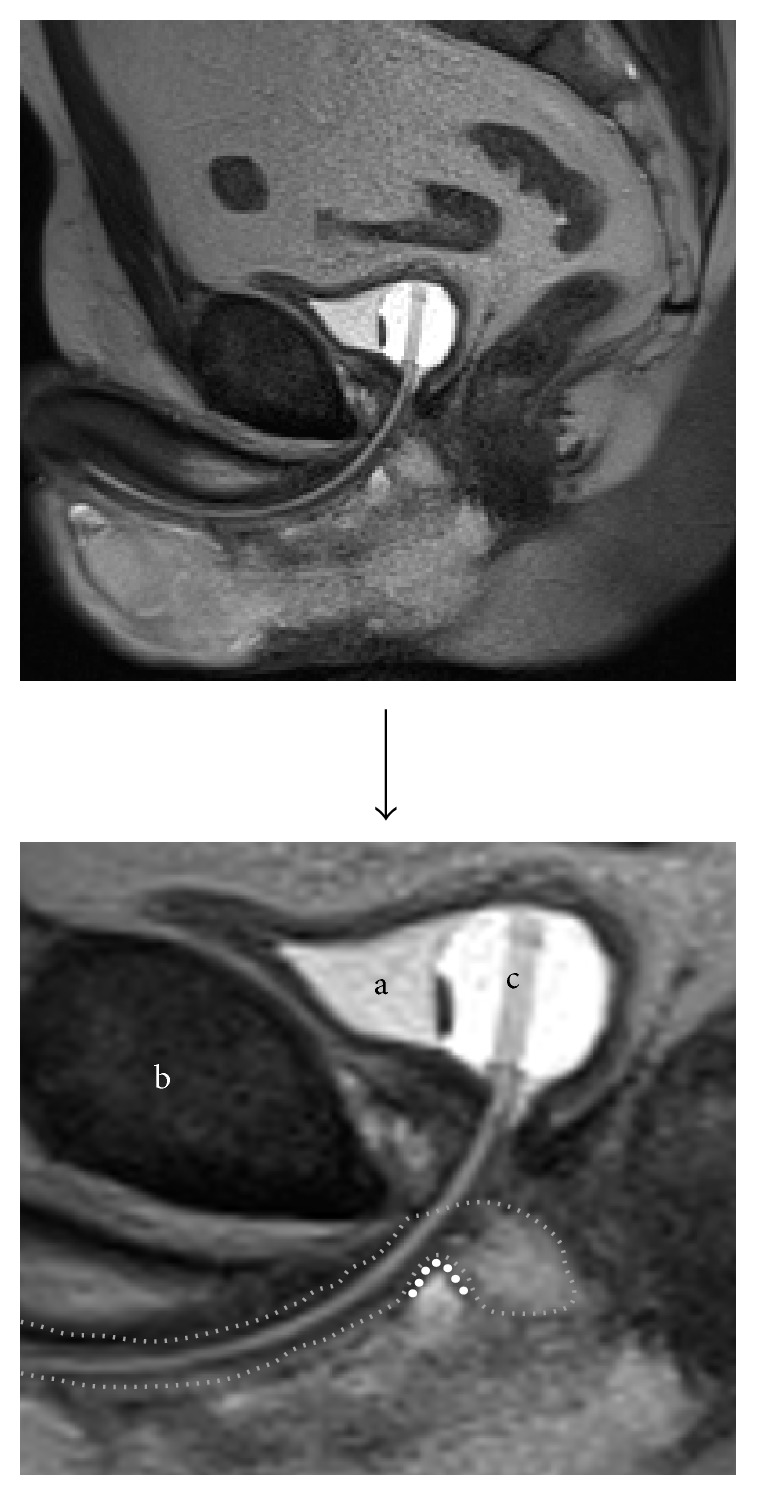
Proximal indentation of urethral bulb (outline thin dotted line) by transobturator sling (thick dotted line). (a) = bladder. (b) = pubic symphysis. (c) = transurethral catheter.

**Figure 12 fig12:**
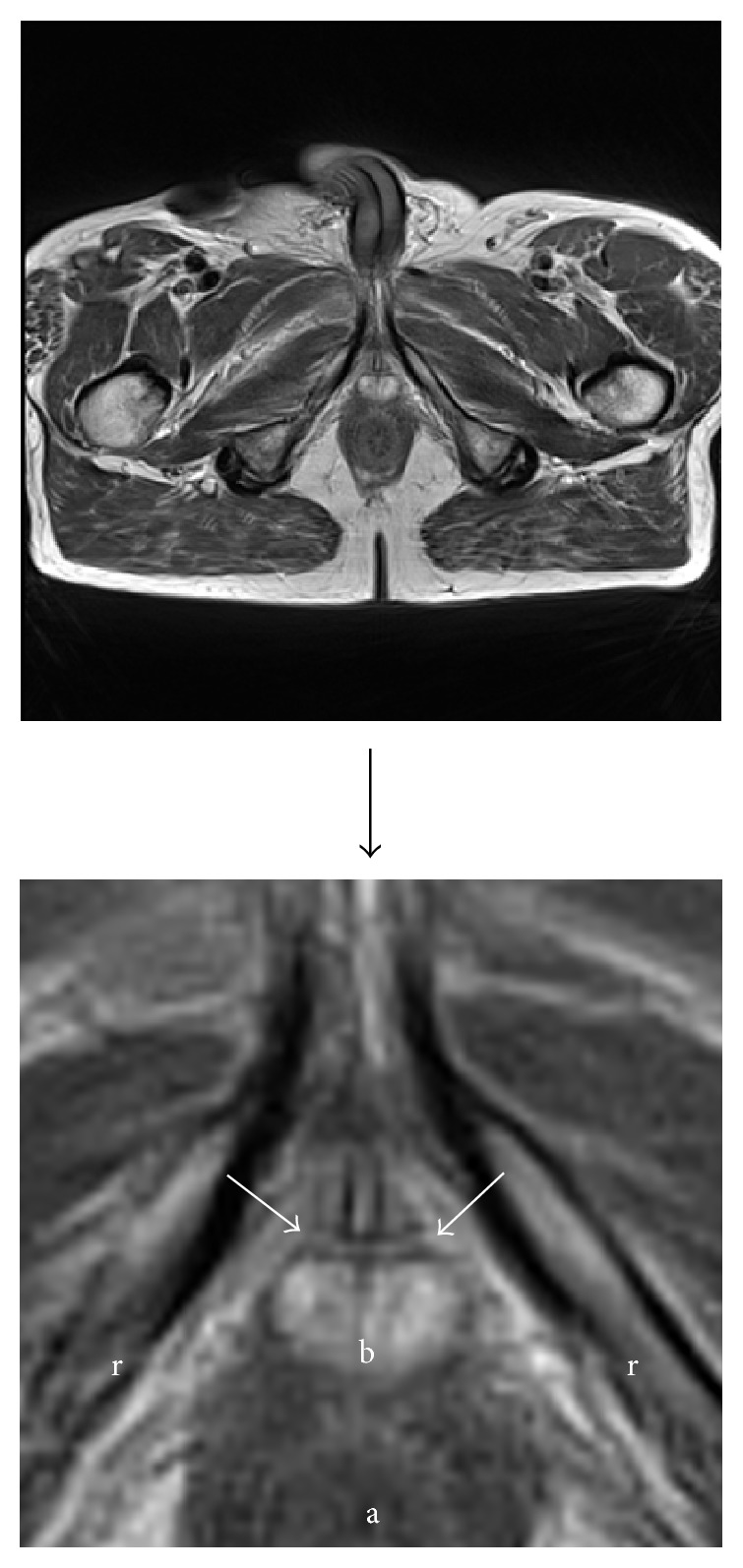
Sling in straight line between inferior pubic rami (lines between arrows). (r) = rami (inferior pubic). (b) = bulb (proximal urethral, posterior urethral bulb). (a) = anus.

**Figure 13:**
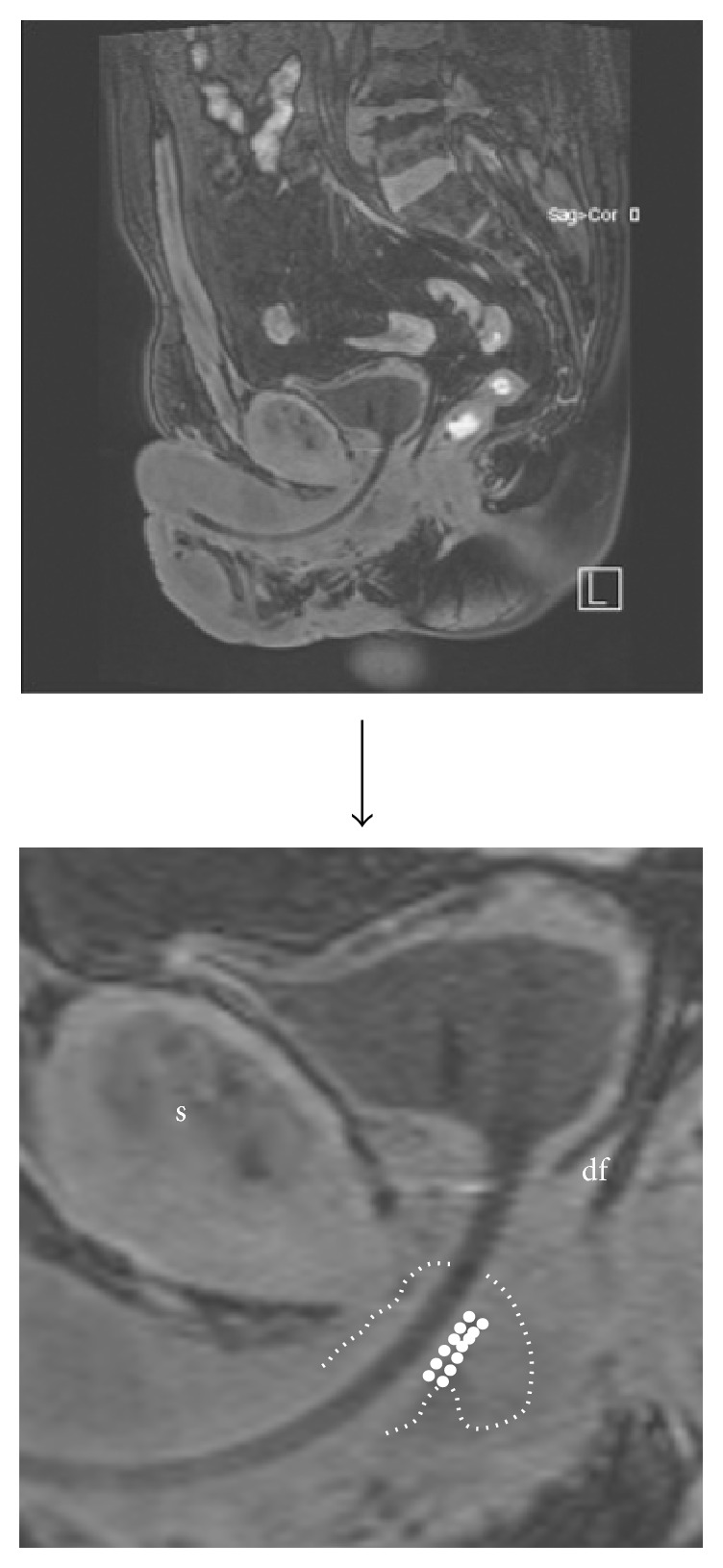
Sling in retroluminal position in double-folded fashion (fat dotted line). (s) = symphysis and (df) = Denonvilliers' fascia. Tightening of the sling leads to a rotational cranial movement with double folding, supporting the dorsal distal aspect of the sphincteric urethra. This support is indirect as spongiosum tissue is interpositioned between sling and urethral lumen. Spongiosum outline indicated with small dotted line.

The length (>10 mm) of the urethral bulb posterior to the sling could be correlated to postoperative continence in patients treated with an AdVance sling [24]. We examined continent patients after sling surgery to demonstrate the ideal sling position. Figures 10 [Fig fig11] [Fig fig12]–13 demonstrate sling position dorsal and caudal to the urethral lumen. Furthermore the bulb is dented showing good vascularity in both proximal and distal aspects. A well vascularized proximal urethral bulb indicates proper proximal blood supply being maintained at the time of prostatic surgery.

### 3.10. Perineal Ultrasound in Postprostatectomy Incontinence and after Sling Placement

**Figure 14:**
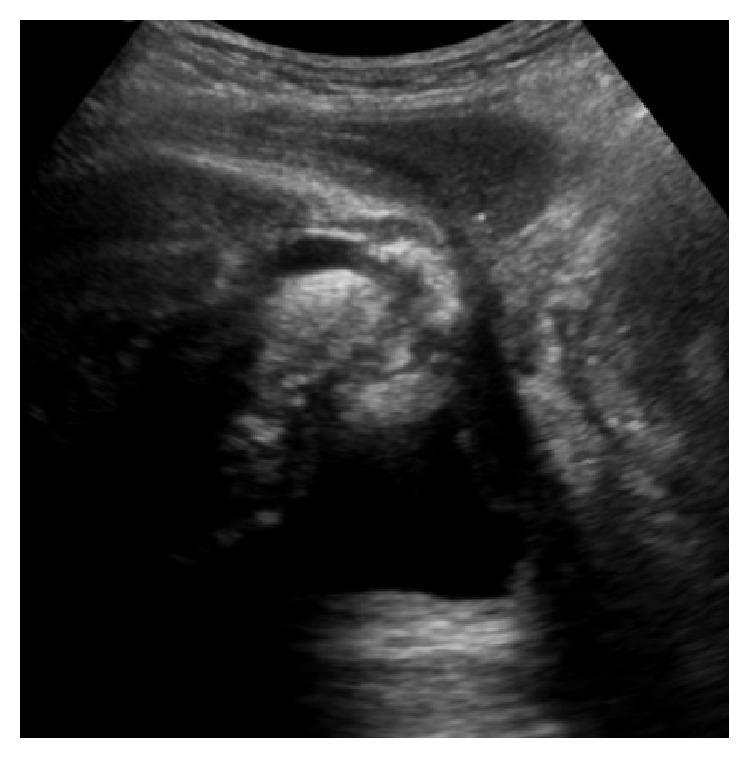
Urethral bulb after radical prostatectomy (no prostate) in patient suffering from urinary incontinence. Explanation: images show perineal skin and bulb top and bladder below (deep), ventrally on left and dorsally on right side as it appears on page; ultrasound probe oriented in midsagittal plane.

**Figure 15:**
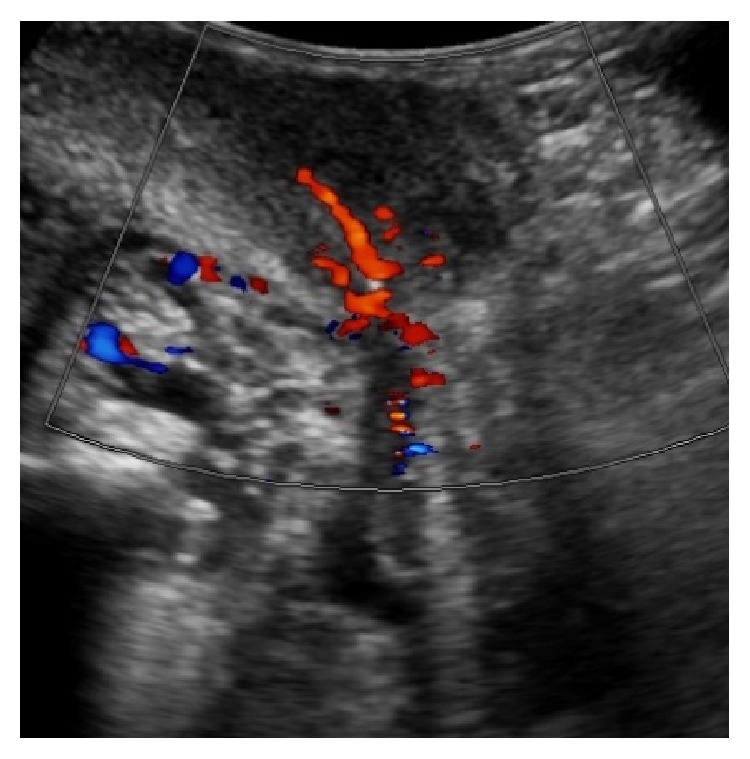
Urethral bulb and prostate in a patient rendered incontinent after transurethral resection of the prostate. Duplex Doppler showing blood flow within proximal bulb and prostate.

Perineal ultrasound may be used to evaluate the urethra, urethral mobility, and opening of the bladder neck during increased intra-abdominal pressure, especially females [25]. It has been shown that the AdVance sling causes dynamic compression of the urethral lumen during increased activity [26]. We wanted to demonstrate sling position on the urethral bulb as well as the maintenance of the blood supply to the proximal and distal corpus spongiosum. Figures 14 and 15 show the extent of the urethral bulb in incontinent patients without and with the prostate still present.

### 3.11. Urethral Bulb Function Relevant to Urinary Continence

The parts of the male urethra from proximal to distal are bladder neck, prostatic, sphincteric (membranous) urethra, pars nuda, bulbous and penile urethra, fossa navicularis, and the meatus. Sphincter fibres are found from the bladder neck down to the beginning of the bulbous urethra. The greatest mass of muscle fibre substance within the sphincter mechanism is most pronounced proximally in the case of the lissosphincter and distally in the case of the rhabdosphincter. The rhabdosphincter anteriorly reaches through the levator ani. Posteriorly the most distal extension of the membranous urethra is enveloped by the corpus spongiosum. The proximal corpus spongiosum is covered by the bulbospongiosus muscle.

Contraction of the bulbospongiosus muscles leads to an increase in pressure within the urethral bulb. This increase of pressure is transmitted onto the lumen of the distal membranous urethra as blood is not compressible. The urethral lumen is thus obstructed to the flow of urine, maintaining continence, also in the presence of maximal physical activity. These mechanisms also facilitate the antegrade propulsion of ejaculate during orgasm.

**Figure 16:**
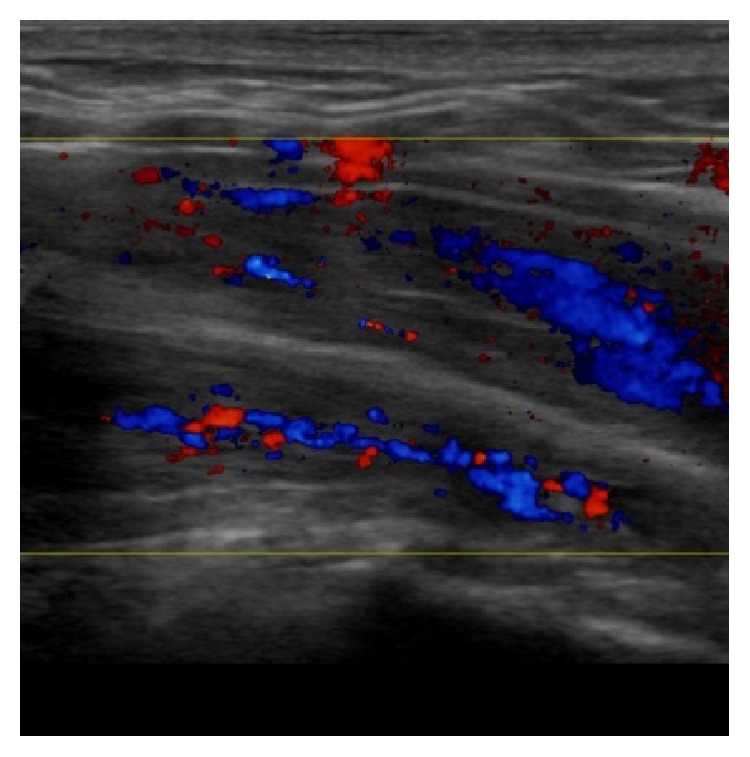
Midperineal elevation test: relaxed state without elevation. Normal blood flow within CS.

**Figure 17:**
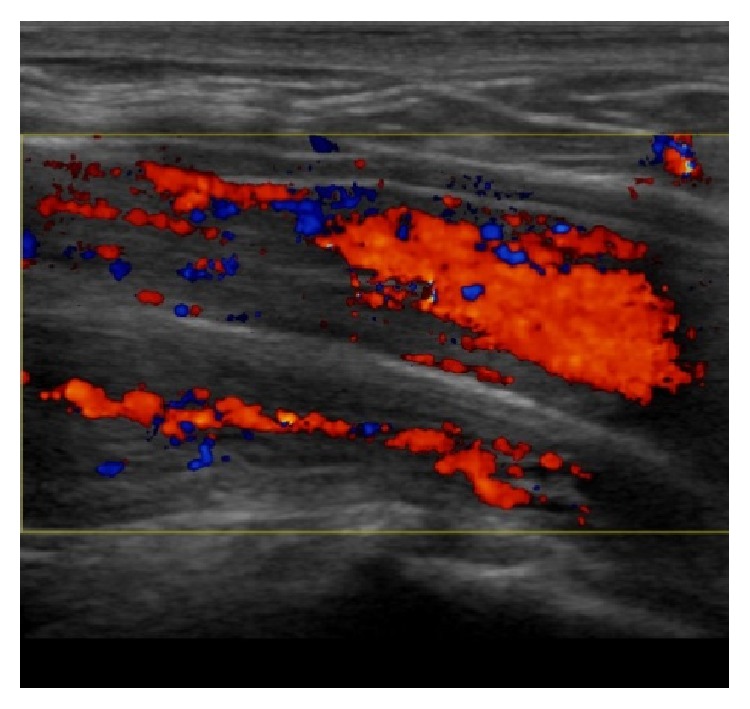
Midperineal elevation test: relaxed state with elevation. Increased Doppler activity during elevation within corpus spongiosum (CS).

Pointed elevation of the midperineum parallel to the membranous urethra and anal canal leads to a response of the distal urethral sphincter mechanism (Figures 16 and 17). A healthy sphincter reacts by concentric luminal occlusion causing lengthwise folding of the urothelial mucosa. This causes a decrease in flow of urine, or an increase in leak point pressure. This reaction lasts to a more of lesser degree as long as the midperineal elevation is maintained. This makes sense as the rhabdosphincter contains mainly slow-twitch muscle fibres.

**Figure 18:**
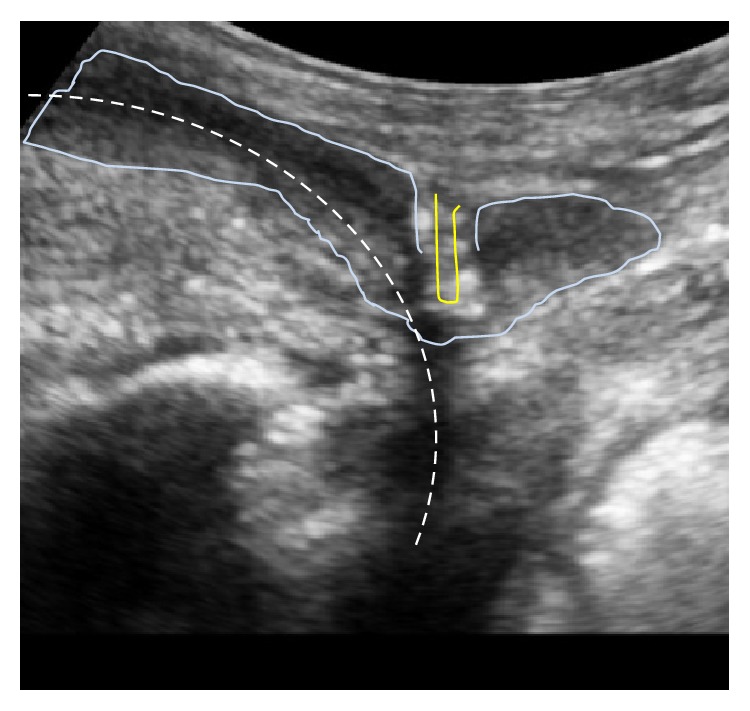
Sling position indenting the urethral bulb. Dotted line indicates path of urethral lumen, double yellow line represents the sling, and light blue outline indicates the indented urethral bulb.

**Figure 19:**
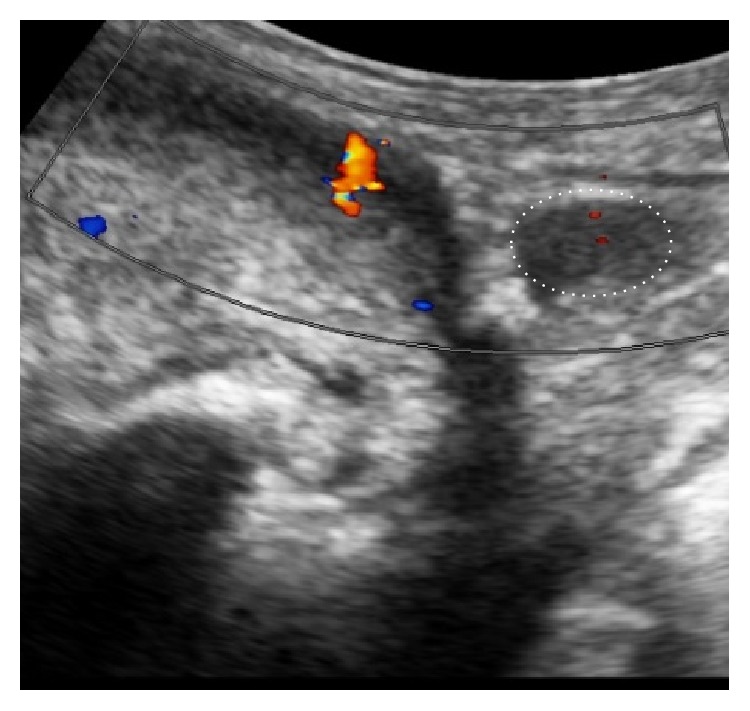
Duplex Doppler showing blood flow after sling placement. Blood flow within urethral bulb after sling placement is maintained. Note the healthy (>1 cm long) proximal portion of urethral bulb on the right side (white dotted ellipse).

The placement of a sling, supporting the distal urethral bulb as described, may be used to treat mild to moderate postprostatectomy incontinence (Figures 18 and 19).

## 4. Discussion

A healthy urethral bulb/corpus spongiosum is of critical importance for the successful outcome of the transobturator retroluminal repositioning sling. Radiotherapy reduces the blood supply to the corpus spongiosum with poor results after sling placement [27–33]. Direct injury to the urethral bulb during sling surgery may also cause damage with resultant scarring and a poorer outcome after sling placement. As shown in Figure 10 the sling clearly lies dorsally to the urethral wall, relying on healthy blood filled spongiosum tissue to help coapt the urethral lumen during increased abdominal pressure. The pressure may thus be transmitted from the retroluminal sling functioning as a dynamic hammock to help treat stress urinary incontinence.

Caremel and Corcos summarize newer techniques in the treatment of male urinary incontinence [5]. It has to be clearly stated that the mechanism of action of the AdVance Male Sling System is different to the compressive nature of other slings or devices. Studies are needed to evaluate the efficacy of these treatments against, for example, the “gold standard,” the AUS AMS 800. When mechanisms of action vary it might be unfair to compare “apples with pears.” The AdVance Male Sling System compares well in terms of clinical outcome, although correct indication for operation is imperative.

## 5. Hypothesis

The proximal male urethral bulb is an integrated part of the urinary continence mechanism, especially during increased physical activity. Sphincter function may be supported by dynamic compression, when it is compromised after prostatic surgery. Such dynamic compression is achieved by placing a transobturator sling to proximally indent the corpus spongiosum. A hammock now exists with a cushion of blood filled spongiosum tissue, to dynamically lengthen the zone of coaptation in the distal sphincteric urethra during moments of increased physical activity.

## 6. Conclusion

The male urethral bulb most probably has an important additional function to maintain urinary continence during physical exercise also in normal men. Contraction of the bulbospongiosus muscles indirectly leads to coaptation of the distal membranous and proximal bulbous urethra. An increased coaptation of the urethral lumen distal to the membranous urethra ensures continence by lengthening the coaptive zone, starting at the bladder neck, throughout the prostatic urethra to the membranous urethra including the pars nuda [34] onto the proximal bulbous urethra.

Diagnostic criteria important for the implantation of a functional sling (transobturator retroluminal repositioning sling) in postprostatectomy urinary incontinence are mild to moderate urinary incontinence, being dry at night in bed, being able to hold back urine when getting up to void, a length of zone of endoluminal urothelial coaptation by >1 cm during dynamic urethroscopy, a healthy looking sphincteric urothelium, and probably good detrusor function.

The AdVance/AdVance XP sling functions by dynamic support of the sphincter mechanism during stress and not primarily by passive compression. The low rate of erosion demonstrates this clearly [6, 24, 28, 35–55]. Most complications were Dindo grade 1. Erosions should not occur; urethral injury is possible though by incorrect operative technique. Slings and devices that obstruct the urethra by compression have significantly higher erosion rates. Ideally an operation to treat male stress urinary incontinence should respect the blood supply to the urethra, sphincter, and corpus spongiosum. That might be one reason why the artificial urinary sphincter remains successful in the long term. The cuff is placed loosely around the proximal urethral bulb and is opened during voiding to minimize outflow obstruction.

It is our belief that a well vascularized urethral bulb plays an important role in the maintenance of normal urinary continence. Furthermore a well vascularized proximal corpus spongiosum may be utilized in functional continence surgery.

## Figures and Tables

**Figure 9:**
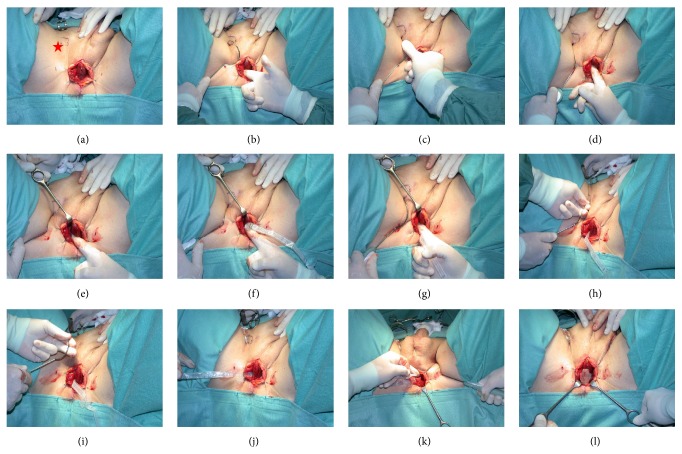
Placement of the AdVance sling [15]: (a) marking the point of entrance for the needle trocar just laterally and below the insertion of the adductor longus tendon (red star). (b) Protecting the urethra with the index finger. (c) The thumb is used to push the helical trocar, in order to deliver the needle tip straight into obturator fossa. (d) The index finger is used to protect the urethra, while rotating the handle of the introducer needle to deliver the needle tip into the perineal wound. (e) The tip of the introducer needle is guided into the perineal wound. (f) The connector attached on the plastic sheath that covers the sling and is clicked into position onto the tip of the introducer needle. (g) The needle tip is pushed back to the edge of the inferior pubic ramus. (h) The helical part of the needle is gripped and jerked a little towards the tip of the ipsilateral scapula, to dislodge it around the broad male inferior pubic ramus. (i) The sling can now be pulled through the obturator fossa. (j) The sling is held out of the way to prepare delivery on the contralateral side. (k) This image shows the sling connector and the helical needle trocar in situ. (l) The sling is pulled into position loosely flushed onto the bulb before fixating the distal edge of the midportion to the bulb with resorbable sutures (Vicryl 2/0).
